# RNA processing mechanisms contribute to genome organization and stability in B cells

**DOI:** 10.1038/s41388-024-02952-2

**Published:** 2024-01-29

**Authors:** Emma Miglierina, Delfina Ordanoska, Sandrine Le Noir, Brice Laffleur

**Affiliations:** 1 University of Rennes, Inserm, EFS Bretagne, CHU Rennes, UMR, 1236 Rennes, France; 2 UMR CNRS 7276, Inserm 1262, Université de Limoges: Contrôle de la Réponse Immune B et des Lymphoproliférations, Team 2, B-NATION: B cell Nuclear Architecture, Immunoglobulin genes and Oncogenes, Limoges, France

**Keywords:** Lymphocytes, Genome

## Abstract

RNA processing includes post-transcriptional mechanisms controlling RNA quality and quantity to ensure cellular homeostasis. Noncoding (nc) RNAs that are regulated by these dynamic processes may themselves fulfill effector and/or regulatory functions, and recent studies demonstrated the critical role of RNAs in organizing both chromatin and genome architectures. Furthermore, RNAs can threaten genome integrity when accumulating as DNA:RNA hybrids, but could also facilitate DNA repair depending on the molecular context. Therefore, by qualitatively and quantitatively fine-tuning RNAs, RNA processing contributes directly or indirectly to chromatin states, genome organization, and genome stability. B lymphocytes represent a unique model to study these interconnected mechanisms as they express ncRNAs transcribed from key specific sequences before undergoing physiological genetic remodeling processes, including V(D)J recombination, somatic hypermutation, and class switch recombination. RNA processing actors ensure the regulation and degradation of these ncRNAs for efficient DNA repair and immunoglobulin gene remodeling while failure leads to B cell development alterations, aberrant DNA repair, and pathological translocations. This review highlights how RNA processing mechanisms contribute to genome architecture and stability, with emphasis on their critical roles during B cell development, enabling physiological DNA remodeling while preventing lymphomagenesis.

## Introduction

Pervasive transcription of genomes, leading to the production of a large variety of noncoding RNAs (ncRNAs), has revolutionized our understanding of RNA synthesis and subsequent RNA processing. RNA processing, as a generic term, refers to the mechanisms that tightly regulate RNA quantity and quality in space and time, by processing and ultimately degrading RNA molecules, ensuring cellular homeostasis. These mechanisms include actors that process RNA molecules through various activities such as unwinding RNA secondary structures, catalyzing RNA modifications, or degrading them from 5′ to 3′ (e.g. XRN1/2 proteins), from 3′ to 5′ (e.g. RNA exosome or DIS3L2), or by cutting directly inside RNA molecules as in the case of endoribonucleases (e.g. RNase H), amongst others [1]. RNA processing is crucial for the quality control and natural turnover of messenger RNAs (mRNAs), but also for the maturation and titration of different ncRNAs including ribosomal RNAs and transfer RNAs. The more recently identified long ncRNAs (lncRNAs), enhancer-associated RNAs (eRNAs), and promoter-associated antisense RNAs (also known as PROMPT-RNAs) can fulfill regulatory functions and are strong RNA processing substrates.

The RNA exosome complex is one of the major RNA processing and degradation factors and plays essential roles in various processes. This complex is ubiquitously expressed, evolutionary conserved in eukaryotes, and is formed by a nine-subunit barrel-shaped core associated with two catalytic subunits, EXOSC10 and DIS3 (in the nucleus) or EXOSC10 and DIS3L (in the cytoplasm). The nuclear RNA exosome ensures the degradation of diverse RNA substrates, including lncRNAs, eRNAs, PROMPT-RNAs, or defective mRNAs. This ribonuclease can associate with cofactors, such as RNA helicases, conferring substrate specificity and ensuring dedicated functions [2–4]. The transcription process, either coding or noncoding, can generate DNA-associated RNAs, also known as R-loops, that are processed by various mechanisms, including RNA exosome-mediated decay. These DNA:RNA hybrids can directly alter chromatin organization, DNA methylation, and chromatin marks [5]. NcRNAs, especially lncRNAs, can also influence chromatin states, usually by acting as scaffolds or bridges for DNA-binding or DNA-modifying proteins [6]. RNA-binding proteins are estimated to represent ~10% of the mammalian proteome and contribute to the regulation of RNA processing and turnover [7]. Recent findings illuminate how RNA processing pathways monitor, regulate, and degrade nuclear RNAs to ensure optimal chromatin organization and associated functions [8]. RNA processing-related mechanisms are frequently deregulated in various pathologies, including cancers, but are also implicated in specific physiological processes, as exemplified with B cells.

B lymphocytes represent a unique model as they express site-specific ncRNAs during their development and orchestrate physiological DNA recombination, but can also undergo pathological DNA alterations that lead to lymphoproliferation. The role of the RNA exosome during early B cell development and later during B cell activation has been well established [9–14]. In multiple myeloma (MM), recurrent mutations of the *DIS3* gene [15], which encodes the major nuclear catalytic subunit of this complex [16], illustrate the importance of these pathways during the ultimate step of B cell differentiation into plasma cells (PCs).

Considering the growing evidence that demonstrates the role of RNAs in maintaining chromatin homeostasis at different levels, we aim to provide here an overview of the mechanisms associated with RNA processing itself that are important for regulating chromatin states and genome organization while preventing genome instability, especially in B lymphocytes.

## The role of RNA processing in genome organization and stability

### RNA processing participates in epigenetic regulations

**Fig. 1:**
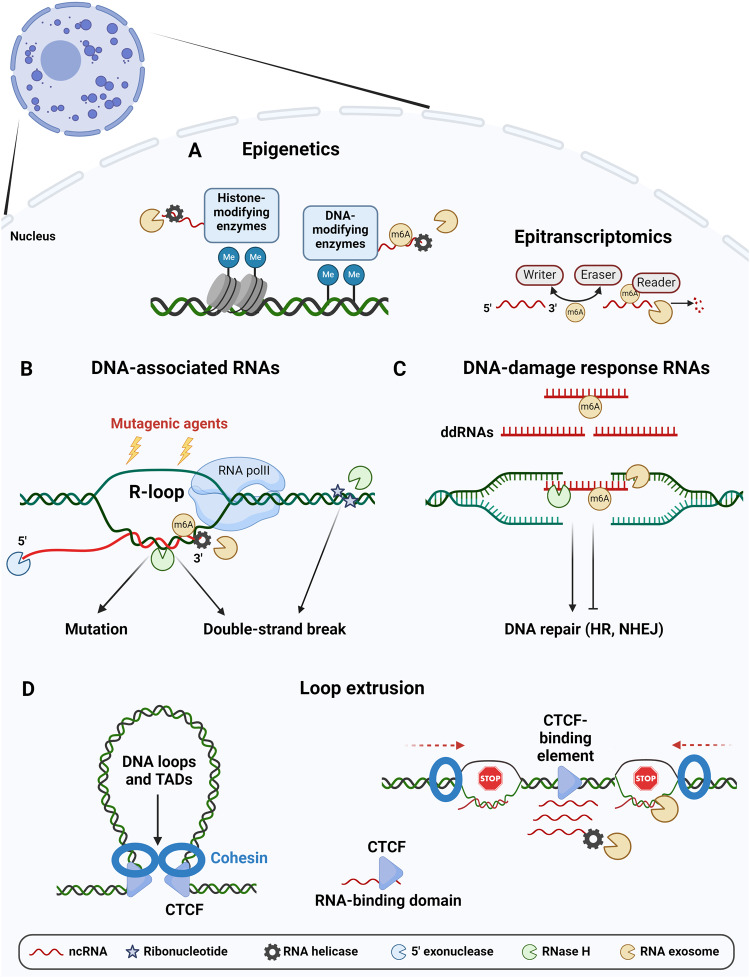
RNA processing mechanisms contributing to genome organization and stability. Eukaryotic genome and chromatin organization are influenced by ncRNAs and RNA processing actors, including 5’ exoribonucleases, endoribonucleases, 3’ exoribonucleases, RNA helicases, RNA-modifying enzymes, and RNA-binding proteins, amongst others. RNA helicases and ribonucleases are particularly important to unwind RNAs and DNA-associated RNAs and ultimately to degrade them. **A** NcRNAs influence chromatin epigenetic states by facilitating the recruitment of histone- and DNA-modifying enzymes such as Polycomb repressive complex or DNA methyltransferases, respectively. RNA processing mechanisms regulate these ncRNAs and thus influence chromatin states. In parallel, RNA-modifying enzymes can catalyse (writer) RNA chemical modifications, such as m6A, remove them (eraser) and bind to them (reader). These RNA modifications modulate the half-life and associated biological activities of different types of RNA transcripts. **B** Transcription can create DNA-associated RNAs, forming R-loops, that are processed by various mechanisms to prevent their deleterious effects on genome stability, such as DNA mutations and double-strand breaks. Ribonucleotides incorporated into DNA also threaten genome integrity and are processed, notably by RNase H. **C** DNA-damage response RNAs (ddRNAs) can be produced after DNA breaks, either facilitating or inhibiting DNA repair by HR or NHEJ. RNA processing pathways contribute to ddRNA regulation and successful DNA repair. **D** Left. The mechanism of loop extrusion relies on the architectural proteins CTCF and cohesin. CTCF proteins directly bind to the DNA while the cohesin complex extrudes DNA to create DNA loops and TADs. Right. The binding of CTCF to the chromatin is dependent on its RNA-binding domain, ncRNAs are produced at CTCF-binding elements and are processed by RNA helicases and the RNA exosome, while cohesin scanning is slowed down by DNA:RNA hybrid accumulation.

NcRNAs can act as scaffolds for the establishment of protein complexes that include DNA methyltransferases and epigenetic writers, readers, and erasers, and for their recruitment to target DNA regions, thus contributing to the regulation of chromatin states and transcription programs [6]. Accordingly, RNA processing mechanisms and RNA epitranscriptomic modifications can also contribute to epigenetic regulations and subsequent functions [17–21] (Fig. 1A).

### RNA processing safeguards genome integrity

Our genomes are constantly subjected to stressors that threaten their integrity at different levels, thus requiring efficient repair systems. RNAs are now proposed to play their part in DNA maintenance, but the intricate relationship between RNAs and genome stability is complex and ambivalent, with examples describing RNAs as actors of DNA repair, while others depict them as threats to genome stability, emphasizing the need for balancing RNA quantity by RNA processing [22]. The first aspect of genome instability occurs at the whole chromosome-scale, where improper RNA processing can lead to aneuploidies and translocations. The contribution of ncRNAs and RNA processing to chromosome integrity is notably related with the regulation of centromeric and telomeric transcripts [23–28]. At the chromatin level, RNA accumulation in R-loop structures can be detrimental, inducing mutations and DNA double-strand breaks (DSBs) [5, 29]. Multiple actors are implicated in the resolution of these structures, of which RNA helicases and ribonucleases, including the RNA exosome [8, 30]. Genetic instability can also be generated through the accidental incorporation of ribonucleotides into DNA, particularly during DNA replication [31]. Accordingly, eukaryotic cells have evolved enzymatic machinery to remove intrusive ribonucleotides, and invalidating mutations in one of their components are pathological, as exemplified by the Aicardi-Goutières syndrome, where RNase H2-deficient cells accumulate DNA-incorporated ribonucleotides and DNA damage [32, 33] (Fig. 1B).

### RNA processing contributes to DNA repair

Oppositely, increasing numbers of studies propose that ncRNAs positively contribute to DNA repair. Noncoding transcription was proposed to be induced locally at DNA breakage sites, and the resulting DNA damage response RNAs (ddRNAs) are suspected to act as recruitment platforms for homologous recombination (HR) and non-homologous end joining (NHEJ) machineries [34]. Diffusible RNA molecules can also be recruited at breakage sites to contribute to damage repair [35]. Of note, the model of RNA-templated HR was proposed in the yeast *Saccharomyces cerevisiae* and could take place in human cells as well [36, 37]. Loss of function of EXOSC10 leads to a local accumulation of DNA:RNA hybrids containing ddRNAs that impair the recruitment of replication protein A on single-stranded DNA and consequently decreases resection during HR [38], suggesting that these ddRNAs are transient actors that ultimately need to be degraded for efficient DNA repair. Similarly, BRCA2 was proposed to recruit RNase H2 at DSB sites to suppress DNA:RNA hybrids that could impair the HR process [39]. In contrast, a study showed an accumulation of RNAs at breakage sites and identified a positive role for RNA methylation by the m6A writer METTL3 in the recruitment of DNA repair factors [40]. RNAs creating R-loops generated at damage sites can also undergo m6A methylation and contribute to HR-mediated DNA repair [41]. The contribution of RNAs to the DNA damage response is seemingly a question of balance, and functional RNA processing appears crucial for efficient repair (Fig. 1C).

### RNA processing is involved in genome organization

Genome architecture relies on multiple levels of organization, starting with DNA packaged around nucleosomes and interacting with DNA-binding proteins to form chromatin. Architectural proteins, including CTCF and the cohesin complex, amongst others, organize chromatin into loops and topologically associating domains (TADs) inside which DNA interactions are more frequent. A/B compartments then delineate active from inactive chromatin while chromosome territories organize the nuclear compartment [42]. At least two non-mutually exclusive mechanisms participate in genome organization: loop extrusion and phase separation. RNA molecules contribute to the nuclear structure at different levels, from global nuclear compartments to local chromatin loops. LncRNAs, like *Xist*, were shown to contribute to the formation and localization of nuclear compartments [43], possibly through mechanisms that imply phase separation [44]. NcRNAs have also been identified as determinant factors for the formation of chromosome territories [45].

During loop extrusion, CTCF proteins directly bind DNA on oriented CTCF-binding elements (CBEs), while the cohesin complex forms a ring that is loaded on chromatin, extrudes DNA, and stabilizes at two CTCF-bound convergent CBEs, creating stable DNA loops and TADs. Finally, the cohesin complex is unloaded by WAPL proteins [46]. CTCF proteins contain an RNA-binding domain that influences their ability to bind DNA [47], while cohesin binds RNA and DNA-associated RNA via its STAG1/2 subunits [48]. There is growing evidence that some ncRNAs, in particular when part of R-loop structures, are involved in the shaping of TADs by loop extrusion [49, 50], and several studies highlighted the importance of R-loop resolution by the RNA processing machinery for efficient loop extrusion. CTCF proteins interact with the RNA helicase DDX55, and together they locally contribute to the regulation of the 3D chromatin structure [51]. A functional interaction implicating CTCF, the helicase DDX5 and its associated ncRNA also contributes to loop stabilization by the cohesin complex [52]. CTCF-associated RNAs have been described in B cells [53], and RNA exosome inactivation induces an accumulation of CBE-overlapping RNAs and a concomitant decrease in CTCF chromatin binding. Furthermore, R-loops accumulate and cohesin localization at CBEs is altered, suggesting that DNA:RNA hybrids slow down cohesin scanning and stabilization at these sites, ultimately leading to genome disorganization [13]. Another study suggests cohesin and TAD boundary loss in the absence of RNA processing by the RNA exosome [54], while R-loops were recently shown to directly slow down cohesin progression, leading to decreased DNA compaction [55]. The kinetics of R-loop formation and resolution by RNA processing therefore appear critical for loop extrusion (Fig. 1D).

Overall, RNAs and RNA processing both contribute to organizing and safeguarding chromatin and genome structure through diverse mechanisms, some of which are particularly important for B cells.

## RNA processing drives B cell development

### Genetic remodeling during B cell development

**Fig. 2:**
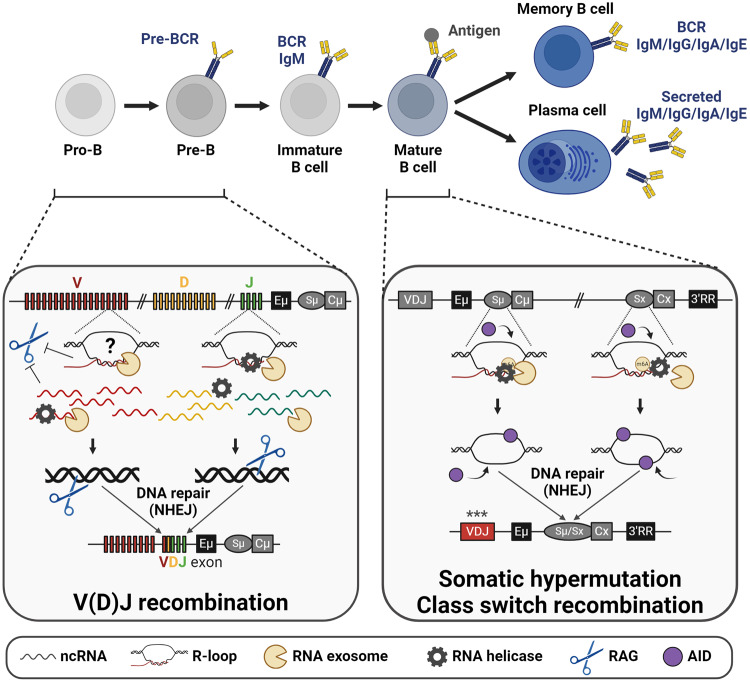
RNA processing contributes to B cell development. B cell development is tightly related with DNA remodeling events, all preceded by noncoding germline transcription at recombination sequences. Left. In pro-B cells, sense and antisense germline transcripts are expressed at V (variable), D (diversity) and J (junction) genes of the *Igh* locus. These ncRNAs are processed by RNA helicases and the RNA exosome for efficient VDJ recombination, mandating pre-BCR expression and pro- to pre-B cell transition. In the absence of RNA processing, these ncRNAs accumulate and strongly affect the quality and quantity of VDJ junctions, abrogating B cell development. Right. In activated B cells, sense germline transcription occurs at donor (Sµ) and acceptor (Sx, x being either γ, α, or ε) switch sequences, generating long R-loops. RNA helicases and the RNA exosome process these DNA-associated RNAs, allowing the generation of single-stranded DNA on both DNA strands, which are bona fide substrates for the AID enzyme. Deamination is then initiated on both DNA single strands, ultimately resulting in DSBs followed by DNA repair for efficient class switching. In the absence of RNA processing, R-loops accumulate, qualitatively and quantitatively altering CSR junctions. A similar phenomenon occurs during somatic hypermutation at VDJ exons, where RNA exosome activity is necessary to degrade sense RNAs for the distribution of AID-mediated mutations on both DNA strands. R-loop accumulation also disorganizes the *Igh* TAD, decreasing the interaction between the critical enhancers Eµ and 3’RR, contributing to reduced CSR but increased aberrant translocations. Figures created with BioRender.com.

B lymphocytes are specialized in the production of membrane-bound B cell receptors (BCRs) that are ultimately secreted by PCs in the form of immunoglobulins (Ig), also known as antibodies, providing humoral immunity. Their development is closely related to ncRNA expression and associated DNA remodeling events [56], including recombination and mutation of their Ig genes, as these loci have a complex germline organization and must be first rearranged to create functional V(D)J exons that correspond to the antigen (Ag) binding site. The V(D)J recombination process relies on the recombination activating gene (RAG-1/2) recombinases that bind and cut DNA before DNA repair joins the V(D)J segments together. A pre-BCR is created after Ig heavy chain (*Igh*) recombination in pro-B cells, while Ig light chain (*Igl*, either κ or λ) rearrangements in pre-B cells allow the expression and the association of the Ig heavy and light chains to form a functional IgM BCR that is expressed at the cell surface. BCR expression is mandatory for B cell maturation and allows their exit from the bone marrow towards the periphery and secondary lymphoid organs. Ag encounter together with costimulatory signals triggers a second wave of gene rearrangements mediated by the activation-induced cytidine deaminase (AID) enzyme during the germinal center (GC) reaction. During this step, B cells divide intensively in the dark zone of GCs to amplify the selected clones and then migrate to the light zone where the best clones are selected for their Ag affinity. Increasing Ag affinity relies on somatic mutations that are introduced by the action of the AID enzyme on V(D)J genes, in a process called somatic hypermutation (SHM). AID also initiates cytidine deamination at switch (S) regions, followed by a series of events leading to DSBs and class switch recombination (CSR), that will change the constant part of Igs from µ to γ, α, or ε, switching the production from IgM to IgG, IgA, or IgE, for recruiting other functions of the immune system. Finally, B cells exit the GC and either differentiate into memory B cells or PCs secreting high-affinity antibodies, ensuring long-term immunity [57] (Fig. 2).

### The early life of B cells

When committed to the lymphoid lineage, B cell precursors initiate V(D)J recombination to create functional Ag binding sites. During this process, Ig genes undergo major epigenetic modifications to become “accessible” for recombination, including nuclear relocation and chromatin modifications. A reorganization of the 3D structure of these loci is also required, as V(D)J segments span over several megabases and must be brought closer together for efficient recombination. 3D reorganization of Ig loci is thus critical for V(D)J recombination and early B cell development, and the role of the transcription factors YY1, PAX5, E2A, and Ikaros has been well documented [58], as Ig loci contain several regulatory elements and enhancers that contribute to their transcriptional regulation, 3D organization, recombination, and SHM [59–61]. Additionally, the architectural elements CTCF, cohesin, WAPL, CBEs, and the associated mechanism of loop extrusion largely contribute to *Igh* locus organization during V(D)J recombination [62, 63]. In contrast, *Igκ* recombination is less sensitive to loop extrusion perturbation but relies on alternative mechanisms of chromosomal folding [64].

According to the current model, before recombination in pro-B cells, *Igh* chromatin becomes accessible, transcription factors and DNA-binding proteins are recruited, inducing noncoding transcription at numerous sites of the locus, including at V_H_ genes. This germline transcription (i.e. transcription from the germline configuration of Ig loci, before recombination) was described a long time ago [65–67], but the fate of these germline ncRNAs had, until recently, remained a mystery. In fact, at this developmental stage, RNA helicases and the RNA exosome are necessary for the processing and degradation of these RNAs, otherwise, the VDJ recombination step is severely compromised and B cell development fails. Mouse pro-B cells lacking the catalytic subunit DIS3 show massive accumulation of sense and antisense ncRNAs at the *Igh* locus. Importantly, these ncRNAs overlap recombination signal sequences (RSS). The introduction of a prearranged VDJ allele at the *Igh* locus rescues the pro-B to pre-B developmental defect, arguing for a direct role of RNA processing in the *Igh* recombination process itself [14]. In humans, mutations of the RNA helicase *SKIV2L* gene perturb RNA processing and lead to trichohepatoenteric syndrome, characterized notably by B lymphopenia. This phenotype is recapitulated in a B cell conditional knock-out mouse model targeting *Skiv2l*, that displays a similar B developmental blockade with V(D)J recombination defects [68], thus directly demonstrating the importance of this RNA helicase during early B cell development. Alterations of RNA processing pathways and their impact on V(D)J recombination underline the necessity to process germline ncRNAs for accurate physiological recombination (Fig. 2). The exact implicated mechanisms remain to be dissected; still, one could hypothesize that ncRNAs accumulate as R-loops at V(D)J genes and RSS sequences, directly impeding RAG binding and cleavage, as these regions could potentially form R-loops and associated G-quadruplexes (G4s) [69, 70]. Chromatin marks could be altered by ncRNA accumulation, especially the important H3K4me3 that facilitates the recruitment of the RAG2 recombinase to RSS targets [71]. An imbalance in RNA quantity and/or quality could also impact RAG1 localization from the RNA-rich nucleolus [72]. Importantly, the loop extrusion mechanism, which is critical for V(D)J recombination [62, 73], could be affected as well. Inactivation of the m6A writers METTL3 or METTL14 also causes an accumulation of pro-B cells [74, 75], underlining the importance of epitranscriptomics during early B cell development.

The V(D)J recombination process is error-prone, implying random usage of the V, (D), and J genes coupled with nucleotides insertion by the terminal deoxynucleotidyl transferase (TdT), and thus generates out-of-frame junctions in two-thirds of the cases. These unproductive junctions induce a frameshift in the open reading frame of the V(D)J exons and the appearance of premature termination codons. When this phenomenon occurs, the second allele undergoes V(D)J recombination, and when successful these cells develop with both productive and unproductive alleles. As the transcription of Ig genes is biallelic, these nonproductive transcripts must be processed by RNA surveillance mechanisms. Splicing inhibition, nonsense-mediated decay, and nonsense-associated altered splicing cooperate to ensure efficient inhibition of these RNAs both at the *Igh* and *Igl* loci during early B cell development and later during PC differentiation [76–79]. It has also been reported that, during DNA repair at V(D)J junctions, ribonucleotides are incorporated into DNA by the DNA polymerase µ and the TdT. The ribonucleotide excision repair machinery, especially RNase H2a, removes these ribonucleotides that are then replaced by deoxynucleotides. Transient ribonucleotide incorporation allows subsequent DNA ligation and successful recombination [80]. This mechanism could be affected by ribonucleotide pool imbalance [81], in particular in cells where RNA processing is compromised. Accordingly, we observed an alteration in CDR3 lengths (corresponding to the V_H_-D-J_H_ junctions) in the absence of RNA processing [14], possibly related to this phenomenon or other DNA repair alterations. Again, a fine equilibrium between RNA metabolism and RNA processing is needed for accurate V(D)J recombination.

### Mature B cell activation

After successful creation of the V(D)J exons and expression of a functional BCR, B cells migrate towards secondary lymphoid organs in search of antigens. During B cell activation, other ncRNAs are produced that, again, must be processed for efficient CSR and SHM.

### Class switch recombination

CSR occurs inside the *Igh* locus between two long repetitive G-rich S regions that are transcribed and produce ncRNAs, known here as switch germline transcripts (GLTs). Furthermore, these ncRNAs create long R-loops at S regions that facilitate the CSR process as they open the DNA double helix, thus exposing the non-template DNA strand to the action of the AID enzyme that initiates recombination [82]. RNA processing actors, including RNA helicases and ribonucleases, are necessary to “resolve” these R-loops by degrading the DNA-associated RNAs. After RNA decay, the two DNA strands of the S regions are left single-stranded, and thus constitute bona fide substrates for AID-mediated deamination, initiating DNA breaks and recombination (Fig. 2). Therefore, defects in RNA processing pathways induce an accumulation of ncRNAs and R-loops that decrease CSR efficiency. MTR4 is a particularly important RNA helicase for unwinding DNA-associated RNAs [83], especially during CSR, thus allowing the RNA exosome to initiate their degradation and warranting successful recombination [12], while other helicases are also involved.

The role of the RNA exosome is critical during CSR, firstly because this complex is necessary to resolve R-loops and to expose the template DNA strand to AID for deamination. The initial demonstration of this mechanism was performed in vitro using DNA:RNA hybrids incubated with the RNA exosome and by monitoring the AID-mediated deamination footprint, which was altered in the absence of RNA decay. Furthermore, inhibition of a core RNA exosome subunit (Exosc3, totally disrupting RNA exosome assembly) decreased CSR in the CH12 murine B cell lymphoma, demonstrating the necessity of RNA exosome-mediated RNA degradation for efficient CSR in a cellular system [9]. Overexpression of RNase H1 in mouse activated B cells also increased the accessibility of AID to the template strand but without increasing CSR efficiency [84]. An alternative, non-mutually exclusive model proposes that S GLTs are processed post-transcriptionally to generate G4-rich “guide RNAs” that facilitate AID recruitment and targeting to S regions [85]. Another study proposes that the RNA helicase DDX1 is necessary to unwind the G4-RNA structures of GLTs, leading to R-loop formation at S regions in *trans*, and ultimately to CSR [86]. Finally, GLT splicing was shown to be necessary for CSR [87]. The implication of all these post-transcriptional mechanisms demonstrates the critical role of RNA processing and decay during this recombination step.

To dissect the role of the RNA exosome subunits during B cell activation, dedicated mouse models have been developed. *Exosc3* targeting confirms the role of the RNA exosome in degrading DNA-associated RNAs at S regions and AID off-target transcribed loci, including oncogenes, and its impact on CSR [10]. Another mouse model targeting *Exosc10* revealed the importance of the degradation of eRNAs by the RNA exosome for optimal 3′ regulatory region (3′RR) function [11], which is critical for CSR [88]. 3′RR activity was proposed to be dependent on another regulatory element, the “lncRNA-CSR” [11], which is particularly important for CSR to IgA [89]. NcRNA expression and decay are therefore critical for orchestrating 3′RR interactions and functions in the *Igh* locus and, as a consequence, for CSR. Finally, a model targeting the catalytic subunit DIS3 demonstrated the critical role of this enzyme for the resolution of R-loops at S regions, and more globally genome-wide. In normal conditions, cohesin is loaded at the *Igh* locus and drives interaction between the Eµ and 3’RR enhancers by loop extrusion, then allowing alignment of the S regions and the creation of an *Igh* TAD prone to CSR for efficient recombination [90–93]. In DIS3-deficient B cells, the accumulation of R-loops inside S regions and the 3’RR perturbs cohesin localization, Eµ/3’RR interaction, and *Igh* TAD formation, contributing to inefficient CSR. The degradation of S GLTs and the resolution of the associated R-loops is thus critical for efficient AID targeting on both DNA strands as well as optimal *Igh* TAD formation during CSR. Translocation sequencing experiments quantifying physiological *Igh* intra-TAD recombination (i.e. CSR) versus pathological inter-TAD recombination (i.e. translocations) also demonstrated a two-fold increase in aberrant translocations in the absence of DIS3 [13]. It is thus possible that altered TAD conformation favors the diffusion and ligation of DSBs between *Igh* and translocation partners, ultimately leading to increased translocations.

Similarly, some RNA helicases are essential to protect B cells from translocations, especially the senataxin which safeguards cells from genetic instability [94], acting redundantly with RNase H2 [95]. Inactivation of MTR4 and senataxin also induces R-loop accumulation and generates asymmetric DNA mutations at Sµ region and at AID off-target genes, including oncogenes [12], arguing for the necessity of clearing RNAs from DNA:RNA hybrids to avoid asymmetric mutagenesis that could be implicated in cancer initiation or development. Furthermore, GLT ncRNAs are methylated by METTL3, and this m6A mark recruits the YTFH2 reader, the MPP6 adaptor, and the RNA exosome for efficient RNA degradation. In the absence of RNA methylation, CSR is decreased but translocations are increased [74].

DNA junction analyses revealed longer microhomologies in cells deficient for RNA processing [13, 74]. This could be due to a shift from classical NHEJ to alternative NHEJ, testifying an alteration in DNA repair, possibly related with R-loop persistence. A recent study highlighted the critical role of the RNA-binding protein HNRNPU for binding both R-loop complexes and classical NHEJ proteins, in an RNA-dependent manner, to facilitate R-loop clearance and DNA repair during class switching. In the absence of HNRNPU, R-loops accumulate and DNA repair is ensured by the alternative NHEJ. The mechanisms involved could implicate liquid-liquid phase separation, as CSR is decreased in the presence of phase separation inhibitors [96]. Globally, R-loop creation and resolution are involved in CSR, and various RNA processing pathways contribute to their regulation.

### Somatic hypermutation

In parallel, SHM occurs at variable V(D)J genes of the *Igh* and *Igl* loci and introduces mutations that are selected to increase Ag affinity. During this process, sense transcription is initiated upstream of the V(D)J exons on the template DNA strand. A first study investigated *Exosc3*-deficient B cells in which the RNA exosome complex assembly is disrupted, which likely inhibits its association with AID [9], and potentially decreases AID recruitment to the V(D)J exons. In this model, a global decrease in SHM efficiency is observed in the J_H_4 intron [10]. By contrast, specific deletion of the catalytic subunit DIS3 maintains a similar level of SHM on a prearranged VDJ exon (B1-8 allele) at the *Igh* locus. In the latter model, the RNA exosome complex could still be formed, associate with AID, and be recruited to this VDJ exon to induce SHM. By analyzing strand-specific DNA mutations, we detected fewer mutations on the template strand, where sense transcription actively occurs and where RNA, as a result of RNA polymerase II stalling [97, 98], can potentially form DNA:RNA hybrids, thus inhibiting AID-mediated deamination and subsequent mutagenesis. Oppositely, over-exposition of the single-stranded non-template DNA resulted in increased mutations in the absence of DIS3 [13]. These data reveal the need for processing sense RNAs at VDJ genes for efficient SHM, in a mechanism that possibly implies R-loops but still remains to be deciphered. R-loops and associated G4s are likely present at V_H_ genes according to predictions by bioinformatic analyses and G4-sequencing [69] or detection by bisulfite sequencing [70], but the demonstration of the existence of such structures during V(D)J recombination and/or SHM and their impact on these phenomena has not been directly established yet.

A model of RNA-templated DNA repair has been proposed for SHM [99] and supposes the existence of RNA intermediates, DNA polymerases that can function with RNA templates and ADAR-mediated RNA editing. Another RNA-templated mechanism could be implicated in DNA insertions observed at the *IGH* locus in humans, especially the *LAIR1* insertions that have been found in the VDJ exons and confer these antibodies functional binding to *Plasmodium falciparum* antigens, while the original genes were not lost, suggesting a gene amplification mechanism, possibly with an RNA intermediate [100]. Other DNA insertions originating from many sites of the genome have then been specifically identified at the *IGH* locus, in multiple donors [101], but the precise mechanism remains to be discovered. How RNA processing could contribute to these mechanisms is unknown. Despite extensive research, the exact mechanism of SHM remains only partially understood and RNA processing pathways contributing to this process have not been exhaustively identified yet; nonetheless, future investigations could solve this conundrum.

### Terminal plasma cell differentiation

The terminal differentiation of B cells into PCs is accompanied by dramatic alterations of their transcriptome, licensing them for high Ig secretion rates. These major transcriptome changes most likely require ad hoc programmed RNA processing. PC differentiation is initiated inside the GCs, or in the frame of extrafollicular reactions, and generates short-lived or long-lived PCs. Long-lived PCs migrate to their niches, including the bone marrow, where they can sustainably secrete antibodies for years or even decades in humans. Before the initiation of the PC differentiation program, B cells undergo activation during which AID activity and other mechanisms can threaten genome stability. As a result, a fraction of activated B cells may undergo mutations and translocations, potentially initiating lymphoproliferation and PC neoplasms, including monoclonal gammopathy of unknown significance (MGUS), smoldering multiple myeloma (SMM), MM, and PC leukemia, while other genetic and epigenetic alterations arise later and contribute to the pathogenesis of MM [102]. MM is characterized by an accumulation, at multiple sites of the bone marrow, of aberrant PCs with high heterogeneity and genomic instability [103]. In this type of cancer, the *DIS3* gene undergoes frequent somatic mutations (~10 to 15% of the patients, depending on the cohorts) leading to loss of function. More strikingly, almost half of the patients have lost one copy of the *DIS3* allele (located on the q13 arm), and globally, *DIS3* mutations and bi-allelic alterations are associated with poor prognosis [15, 104–109]. *DIS3* somatic mutations have been shown to disturb RNA metabolism of myeloma cells, inducing ncRNA accumulation [110]. DIS3 has also been recently identified as an important factor for the cell cycle progression of MM cells and for preventing centrosome amplification, a mechanism potentially related to genetic instability [111]. Reminiscent of mouse models [13], DIS3-deficient MM patients have more frequent translocations [107], and a recent study also demonstrated that *DIS3* inactivation induces genome instability by increasing the frequency of R-loops in MM cell lines [112]. Interestingly, *DIS3* variants were also detected as germline alterations that decrease DIS3 catalytic activity and predispose patients to develop familial MM [113]. While it is still unclear at which stages *DIS3* alterations happen and how they impact MM initiation and/or development, they are frequently found in MGUS, SMM, MM, and PC leukemia [107, 114–116], suggesting a major contribution to these pathologies that specifically impact terminally differentiating B cells.

Other RNA processing pathways are also affected in MM, including the RNA-binding protein AATF/Che-1, which is another factor contributing to R-loop resolution. By interacting with the lncRNA *NEAT1/2* and with paraspeckles, AATF/Che-1 contributes to R-loop clearance, thus preventing their diffusion to the cytoplasm of MM cells and the activation of the interferon response [117, 118]. The non-canonical RNA poly(A) polymerase TENT5C, which can be mutated in MM, was shown to stabilize mRNAs and gene expression in myeloma cell lines, increasing cell viability [119]. The spliceosome component SF3B1 is altered in some patients and perturbs alternative splicing [120]. *DIS3* and *TENT5C* bi-allelic alterations have been identified to increase alternative splicing, and interestingly some transcripts of the NHEJ pathway are alternatively spliced in MM [121]. The *ADAR1* gene is frequently amplified in this pathology [122], inducing RNA hyper-editing (adenosine-to-inosine) and contributing to poor patient survival, notably by increasing cell proliferation [123]. Finally, RNA modifications are also likely implicated in MM, and the m6A mark was already shown to impact MM cell proliferation and invasion [124].

Overall, B cell development is tightly linked with ncRNA production, processing, and degradation. These RNAs are produced at different regions of the genome but importantly at recombination sites, they are then unwounded and degraded by RNA processing actors to allow the completion of physiological recombination processes. Alterations of some of these mechanisms are found in MM cells, likely contributing to the pathogenesis of the disease.

## Conclusion

RNAs are now considered major players in chromatin remodeling, genome organization, and stability. By regulating a wide variety of RNAs, RNA processing mechanisms directly or indirectly contribute to the fine-tuning of chromatin conformation, higher levels of 3D genome organization, and protection against genome instability. Recent evidence highlights these processes, and their biological relevance is exemplified during B cell development and associated pathologies. Future studies will more precisely and definitively determine the critical contribution of RNA processing in nuclear architecture and genome stability in B cells.
